# The Dorsolateral Prefrontal Cortex Plays a Role in Self-Initiated Elaborative Cognitive Processing during Episodic Memory Encoding: rTMS Evidence

**DOI:** 10.1371/journal.pone.0073789

**Published:** 2013-09-05

**Authors:** Colin Hawco, Marcelo T. Berlim, Martin Lepage

**Affiliations:** 1 Department of Neurology and Neurosurgery, Douglas Mental Health University Institute, McGill University, Montreal, Quebec, Canada; 2 Department of Psychiatry, Douglas Mental Health University Institute, McGill University, Montreal, Quebec, Canada; University of Granada, Spain

## Abstract

During episodic memory encoding, elaborative cognitive processing can improve later recall or recognition. While multiple studies examined the neural correlates of encoding strategies, few studies have explicitly focused on the self-initiation of elaborative encoding. Repetitive transcranial magnetic stimulation (rTMS), a method which can transiently disrupt neural activity, was administered during an associative encoding task. rTMS was either applied to the left dorsolateral prefrontal cortex (DLPFC) or to the vertex (a control region not involved in memory encoding) during presentation of pairs of words. Pairs could be semantically related or not related. Two encoding instructions were given, either cueing participants to analyze semantic relationships (cued condition), or to memorize the pair without any specific strategy cues (the self-initiated condition). Participants filled out a questionnaire regarding their use of memory strategies and performed a cued-recall task. We hypothesized that if the DLPFC plays a role in the self-initiation of elaborative encoding we would observe a reduction in memory performance in the self-initiated condition, particularly for related. We found a significant correlation between the effects of rTMS and strategy use, only in the self-initiated condition with related pairs. High strategy users showed reduced performance following DLPFC stimulation, while low strategy users tended to show increased recall following DLPFC stimulation during encoding. These results suggest the left DLPFC may be involved in the self-initiation of memory strategy use, and individuals may utilize different neural networks depending on their use of encoding strategies.

## Introduction

There has recently been a growing interest in the role of the frontal lobes in long-term memory formation. Neuroimaging studies of item memory encoding have often observed greater activity in the ventrolateral prefrontal cortex (VLPFC) for items which are later remembered over those which are not later remembered [1,2]. Although dorsolateral prefrontal cortex (DLPFC) activity has not been consistently shown in neuroimaging studies of subsequent memory for items, studies examining associative memory (remembering the relationship between two or more items rather than the items themselves) have often reported both VLPFC and DLPFC activity for successful associative memory formation [3–5]. Interestingly, activity in the DLPFC is increased for associative memory encoding compared to item encoding independent of subsequent memory performance [6].

The DLPFC has been proposed to be highly involved in conscious planned control of behavior and cognition [7]. Given that associative encoding may involve greater elaboration or executive control than item encoding, Blumenfeld and Ranganath [4] examined this issue utilizing an effortful, elaborative encoding task for both item and associative encoding. They observed greater activity in the DLPFC for remembered over forgotten stimuli for associative encoding only, while VLPFC activity was greater for subsequently remembered stimuli during both item and associative encoding. This suggests that DLPFC activity was not simply the result of increased task demands and elaboration but was instead related to forming associations between the items.

Additional evidence regarding the role of the prefrontal cortex in memory comes from prefrontal cortex (PFC) lesion studies in humans. PFC lesions can lead to deficits on tests of free recall [8–12] and cued recall [13] usually without the severe amnesia associated with medial temporal lesions [14,15]. While specific localization of memory deficits in PFC lesions is complicated by the heterogeneity of lesions, the most severe memory impairments are often observed following damage to Brodmann’s area (BA) 46 and 9 (the DLPFC) and BA 44 (part of the VLPFC) [16]. Interestingly, people with PFC lesions can exhibit a deficit in self-initiating elaborative and effective memory encoding strategies related to their memory problems. For example, people with prefrontal lesions do not typically engage in semantic clustering [10,17–20], even though this is an effective memory encoding strategy, yet they are capable of performing semantic clustering when explicitly instructed to do so [19,20]. In contrast, healthy individuals will tend to spontaneously utilize elaborative encoding strategies during memory tasks. When examining verbal stimuli, some possible memory strategies include rote-repetition, binding the word into a semantic context, building a sentence from the words, or using mental imagery. Generally, the use of more elaborate and efficient encoding strategies has been linked to better memory performance in healthy individuals [21–23].

Repetitive trans-cranial magnetic stimulation (rTMS) allows a more causative approach in that a functional hypothesis for a specific cortical region can be tested. In healthy participants, rTMS to the left DLPFC during memory encoding has been shown to reduce memory performance for complex scenes [24–26], for unrelated (but not related) word pairs [27,28], and for word lists [29]. Reduction in memory performance following left DLPFC stimulation is similar for both verbal and non-verbal material [30]. However, laterality effects of rTMS stimulation of the DLPFC have been observed between encoding and recognition with left-side stimulation generally reducing performance during encoding and right-side stimulation reducing performance during retrieval or recognition [24–28], suggesting the effects of rTMS on memory are not due to a general disruption of cognition. The results from rTMS studies provide compelling evidence that the left DLPFC is involved in encoding operations during a variety of conditions, rather than principally during associative encoding as suggested by the fMRI literature [3,5].

As mentioned earlier, another possible role for the DLPFC during encoding operations may be in the use of elaborative encoding strategies. The DLPFC is associated with high level cognitive functions and findings in people with prefrontal damage indicate a reduction in the self-initiation of effective elaborative encoding strategies. At present, only a single rTMS study on recognition memory has explored the role of memory strategy [31], with individuals who failed to use memory strategies to aide recognition performance showing different effects from TMS stimulation as opposed to strategy users. Previous fMRI studies on strategy use have typically focused on the neural correlates of specific strategies (e.g. visualization vs. verbalization) rather than the self-initiation process per se.

We recently explored this hypothesis in an fMRI study designed to specifically explore the neural correlates of the self-initiation process [32]. As the goal of this study was to examine self-initiation of elaborative encoding, we utilized two separate encoding instructions that oriented participants towards externally-cued or self-initiated elaborative encoding. Participants were presented triads of objects with varying numbers of semantic relationships (with zero, one, or both of the bottom objects being related to the top objects), and were asked either to evaluate these semantic relationships (for externally-cued strategy use) or to perform a judgment of the relative size of the pictured objects in real life (in which case any elaborative semantic encoding was self-initiated). While there was still some need to “initiate” semantic analysis in the cued condition, they key difference in these conditions was that in the self-initiated condition, any semantic analysis which was performed was not in response to an external cue but instead internally self-initiated, even though it was not strictly necessary to complete the task. In this way, we were attempting to examine the system which is damaged in patients with frontal lobe lesions, who fail to self-initiate encoding strategies but will utilize them when instructed to do so. The left DLPFC and bilateral supramagrinal gyrus were found to have greater activity for both self-initiated over externally-cued encoding and greater activity for semantically related over unrelated triads. These results were interpreted as evidence that the left DLPFC was involved in self-initiating elaborative semantic encoding, while the supramarginal gyrus may have played a role in orienting attention towards the semantic relatedness of the triads.

While our previous fMRI study [32] has suggested the left DLPFC plays a role in the self-initiation process, fMRI is a correlation research technique. In order to establish more causative evidence, we have performed an rTMS study. For simplicity in study design and interpretation, we utilized pairs of words which could be either related or unrelated rather than triplets of objects, and again presented encoding instructions representing either externally-cued or self-initiated elaborative semantic encoding. If the DLPFC does indeed play an important role in the self-initiation of elaborative encoding, we would expect to see a reduction in memory performance following left DLPFC rTMS stimulation during the self-initiated condition mainly for related pairs, and no rTMS effects in the externally-cued condition. In contrast, if the DLPFC plays a more general role in encoding associations, we expected a generalized decrease in memory performance regardless of encoding condition. Furthermore, as we are hypothesizing that the DLPFC is involved in memory strategy use, we collected self-report data on the use of memory strategies, which can then be related to the effects of rTMS on memory. If the left DLPFC is playing a role in self-initiated strategy use, participants who make greater use of memory strategies may show a greater reduction in memory performance following left DLPFC stimulation.

## Methods

### Ethics Statement

All research was conducted according to the guidelines laid out by the Declaration of Helsinki, and was approved by the Research Ethics Board of the Douglas Mental Health University Institute. All participants signed informed consent forms prior to engaging in any study related activity, and were free to withdraw from the study at any time without loss of compensation.

### Participants

Only right handed participants between the ages of 18 to 35 who had native level proficiency in English, and no self-reported history of neurological or psychiatric disorders (including any history of seizures) were eligible to participate in this study. Forty participants were recruited for this study (16 males; mean age 22.8 ± 3.6). Participants were screened for TMS contraindications (such as the presence of metallic objects in the body) and signed an informed consent form prior to the experiment. Of the forty participants recruited, one chose not to complete the experiment due to discomfort during rTMS and four other participants were excluded due to extremely poor performance on the cued recall task (mean recall rate of 3.5%, 4.2%, 6.3%, and 6.9% for these individuals; see below for task details), resulting in 35 participants with usable data.

### Stimuli

Experimental stimuli consisted of pairs of related and unrelated words. All words were concrete visualizable nouns. In order to classify word-pairs as related, norms were collected from a group of 10 English speaking participants. Participants were shown a pair of words on a laptop, and rated the level of relatedness of each pair on a 7-point scale, with 1 being totally unrelated (e.g. hammer - apple), and 7 being very highly related (e.g. hammer - nail). Word pairs with mean relatedness scores above 4.5 and a standard deviation of less than 2.5 were considered to be semantically related. One hundred forty-four word pairs were created for the experiment (72 related and 72 unrelated).

### Experimental Task

The experimental task was performed on an IBM laptop with a 17″ screen, positioned approximately 0.7 meter from the participant’s eyes, using E-Prime 2.0. The experiment was divided into three parts: an encoding phase (during which rTMS stimulation was administered), a questionnaire assessing encoding strategies, and a cued-recall test.

### Encoding Phase

Participants were presented an encoding instruction for 2 seconds, followed by a pair of words with the previous instruction for 2 seconds, and finally a fixation cross for 8 seconds between trials. rTMS was administered while the word pair was on the screen (see 2.4). Encoding instructions oriented participants towards either externally cued elaborative encoding (the 'cued' condition) or self-initiated elaborative encoding (the 'self-initiated' condition). For the cued condition, the task instruction was 'related?'. Participants were instructed to indicate if the presented words were semantically related or not. For the self-initiated condition, participants were shown the task instruction 'memorize' and asked to make a button press once they read the words. We chose the non-specific instruction 'memorize' to ensure participants were free to utilize encoding strategies without an external prompt to do so (so any elaborative encoding was entirely self-initiated). It was emphasized to the participants there would be a memory test later on and they would be tested on all of the word pairs (regardless of whether the task instruction was 'related?' or 'memorize'). So, the only difference between the 2 encoding conditions was if participants were explicitly instructed to judge relatedness (an effective memory strategy) or if they had to self-initiate semantic processing. If participants did in fact use semantic processing during the self-initiated condition, we hypothesized there would be a greater recall for related over unrelated word pairs.

Each word pair was associated with the externally cued encoding condition in half of participants and the self-initiated condition in the other half. The encoding phase was divided into two consecutive blocks of 72 trials (corresponding to the two rTMS blocks; DLFPC and Vertex stimulation) of 12.5 minutes in length.

### Memory Strategy Questionnaire

Immediately after the encoding phase, participants were instructed to fill out a memory strategy questionnaire. Participants were informed that there were four types of trials during the experiment. For each of these separate conditions, they were asked to rate how often they used each of five different memory strategies on a numerical 7-point scale (with *1* corresponding to never, and *7* with always), resulting in a total of 20 items on the questionnaire. These strategies were derived by considering other studies examining memory strategies [33–36], and were selected to provide a good overall measure of participant’s strategy use (allowing for the possibility that individuals may utilize different patterns of strategies while still being overall high or low strategy users). The five memory strategies were:

1 I considered how the words could be related to each other.
2 I imagined the objects described by the words interacting in some way.
3 I used prior personal memories associated with the objects.
4 I constructed a sentence with the two words.
5 I repeated the words to myself in my head.

Questions 1 to 4 can be considered elaborative encoding strategies, in that they include additional cognitive processing related to the stimuli. Question 5 is an alternate memory strategy which may be used by some or all participants, but does not significantly involve elaborative encoding per se. Question 5 can be considered a control strategy, as we do not expect DLPFC stimulation to affect repetition (as it is not an elaborative encoding strategy). Each strategy was briefly explained to the participants.

### Cued Recall Phase

Recall began 30-35 minutes after the end of the second rTMS encoding block, to allow time for any potential carry-over effects of rTMS to wear off. Participants were presented a single word on the computer screen and instructed to indicate which word was paired with the presented word during the encoding phase. Participants responded verbally and then pressed the spacebar to proceed to the next trial. Participants were told to say ‘Pass’ if they were unable to recall the match to the presented word. Responses were coded as correct, incorrect, or pass. While no rTMS was presented during the cued recall phase, it is important to note that cued recall performance is a measure of how well stimuli were encoded. As such, the cued recall score will be used to measure the effects of rTMS during encoding, as decreased cued recall performance would indicate a disruption during memory encoding.

### rTMS Stimulation

High frequency rTMS was administered during the encoding phase using a Magstim Rapid2® magnetic stimulator (Magstim Company Ltd., U.K.) with a focal 70-mm figure-of-eight coil. The resting motor threshold was determined over the left primary motor cortex using the visualization method [37] and the maximum likelihood strategy [38]. Coil positioning was determined by the 10-20 EEG system, such that F3 corresponds to the left DLPFC [39,40] and the vertex corresponds to Cz. For DLPFC stimulation, the coil was placed flat against the scalp with the handle pointing 45° away from the midline; for vertex stimulation, the handle was pointed behind the participant with the coil flat on the head and the handle facing the participant’s back. During word presentation, a 2 second train of 10 Hz rTMS was presented at the resting motor threshold, with a 10 second inter-train interval. Two bursts of rTMS were presented prior to the onset of the first word-pair to acclimatize participants.

Two separate blocks of rTMS were administered for each participant: one block stimulating the DLPFC and the other block stimulating the vertex (as a control). The vertex has been used as a control site in other memory rTMS studies to account for non-specific TMS effects (somatosensation and noise) and memory performance; vertex stimulation has been found to be similar to a no TMS baseline condition [26,41]. The order of rTMS blocks (DLPFC and Vertex) was counter-balanced across participants with half of the participants receiving the DLPFC block first and half receiving the vertex block first. The total number of rTMS pulses in each block was 1440, with all rTMS parameters falling within established safety guidelines [42].

## Results

### Encoding Phase

Encoding accuracy data was only analyzed for the cued encoding task (the 'related?' task instruction), as participants performed a judgment task which could be considered correct or incorrect. The mean accuracy for the cued encoding task was 93.4% for DLPFC trials and 91.3% for vertex trials. No significant difference in accuracy was found for relatedness, rTMS block, or rTMS by relatedness interaction (all p > 0.1). This indicates that TMS stimulation did not interfere with participant’s ability to analyze semantic relationships.

### Questionnaire Data

Questionnaire data was highly skewed (positive for questions 1, 2, and 5; negative for questions 3 and 4), so non-parametric statistics were used. In order to insure there was no effect of the order of rTMS stimulation blocks, participants were separated into 2 groups according to the order of encoding blocks (DLPFC followed by vertex, or vice-versa) and then we compared the mean response for each question within the four types of trials. There was no significant difference in either the gender or age of participants in each group (p = 0.13 for age, independent samples t-test, p = 0.33 for gender, chi-squared test). Data were analyzed using Mann–Whitney U-tests. No significant effect of group was observed for any question (all p > 0.1), indicating it was reasonable to collapse across these groups.

**Figure 1:**
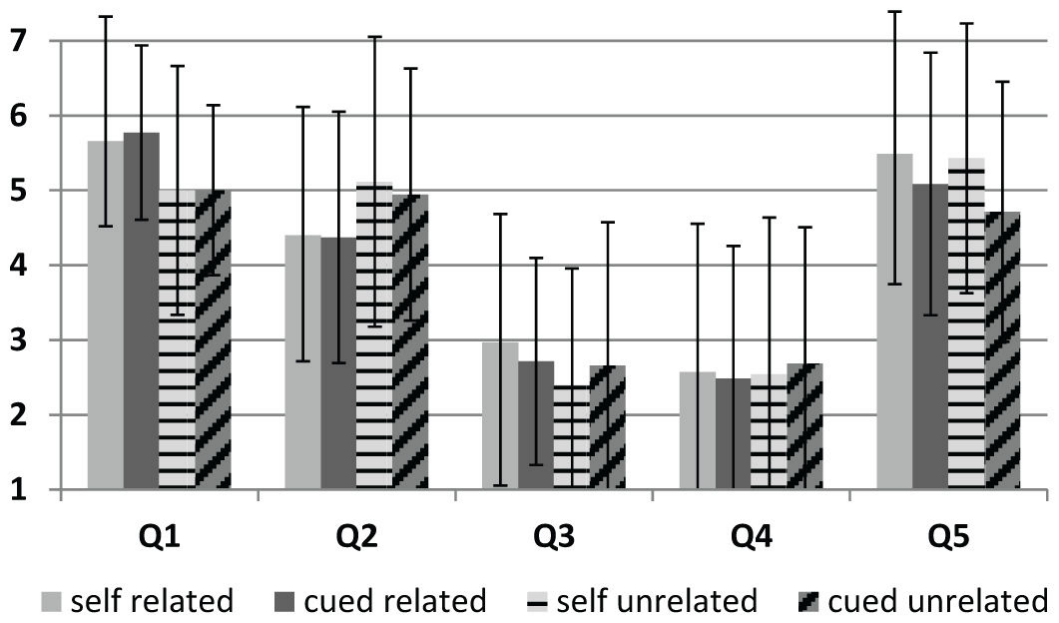
Mean (and standard deviations) of responses given on the memory strategy questionnaire. Participants reported their strategy use on a 7 point scale, with 1 representing “never” and 7 representing “always”. The questions were: Q1. I considered how the words could be related to each other. Q2. I imagined the objects described by the words interacting in some way. Q3. I used prior personal memories associated with the objects. Q4. I constructed a sentence with the two words. Q5. I repeated the words to myself in my head.

The mean response to each question is presented in Figure 1, and the full set of responses for each question for each participant is included in Table S1. To test for differences in strategy use between the TMS conditions, a Friedman’s Analysis of Variance by Ranks was performed. This analysis was applied to each question separately. A significant difference across conditions was found for Question 1 (relatedness), p = 0.023, Question 2 (visualization), p = 0.027, and for Question 5 (repetition), p = 0.010, but not for Question 3 (personal relevance), p = 0.205, or Question 4 (sentence generation), p = 0.958. The results from Question 1 and 2 suggest participants utilized semantic relatedness strategies more for related stimuli but visualization more for unrelated stimuli. The results of question 5 suggest repetition was utilized less in the cued condition.

### Cued Recall Results

Mean cued recall results for each condition are shown in table 1. Percent correct for each condition for each individual (the full data set used in the analysis) is presented in the Table S2. For each of the four conditions in the study, a difference score was calculated to determine the effects of rTMS applied over DLPFC versus vertex. The difference score was defined as subsequent cued recall percent correct for the trials presented in the DLPFC block minus percent correct from the trials presented in the vertex block (such that a negative score indicated reduced performance in the DLPFC block), separately for each condition for each participant. The resultant difference score followed a normal distribution. As such, a 2 x 2 ANCOVA (encoding condition by semantic relatedness) was performed. Given our interest in relating our results to the use of encoding strategy, and the results of [31] suggesting differences in the effects of rTMS based on strategy use in recognition memory, we included mean elaborative strategy use as a covariate of interest. Mean strategy use was defined as the mean of the elaborative encoding strategies (questions 1 to 4), collapsed across the four conditions, on the strategy questionnaire. The results of this ANCOVA showed no significant effects of encoding conditions, F(1,33) = 2.0, p = 0.164, or relatedness, F(1,33) = 0.236, p = 0.63, but a significant interaction between encoding and relatedness, F(1,33) =7.5, p = 0.010. Furthermore, there was a significant interaction between strategy use and encoding and relatedness, F(1,33) = 6.74, p = 0.014. Given the significant interaction, a series of post-hoc paired t-tests were performed on the difference scores from all conditions to identify specific significant differences, resulting in 10 paired t-tests to account for all possible combinations. None of these post-hoc t-tests were significant (all uncorrected p > 0.1). This suggests that the observed interaction is primarily driven by strategy use.

**Table 1:** Cued recall scores, mean (standard deviation).

	**Self-Related**	**Cued-Related**	**Self-Unrelated**	**Cued-Unrelated**
**DLPFC Block**	34.3 (20.1)	40.1 (16.9)	12.2 (14.3)	9.8 (12.6)
**Vertex Block**	35.4 (18.7)	42.1 (15.9)	13.5 (15.9)	7.3 (12.3)
Difference *	-1.1 (21.1)	-2.0 (18.4)	-1.3 (12.7)	2.5 (9.5)

* Difference score is the percent correct of the DLPFC block minus the vertex block for each participant, such that a negative value indicates lower percent accuracy for the DLPFC block.

For completeness, we also performed a similar MANCOVA using Question 5 (repetition) as a covariate to determine if repetition strategy use would have a similar effect on performance; no significant effects were found (all p’s > 0.1).

### The Relationship between Strategy Use and the Effects of rTMS

**Figure 2:**
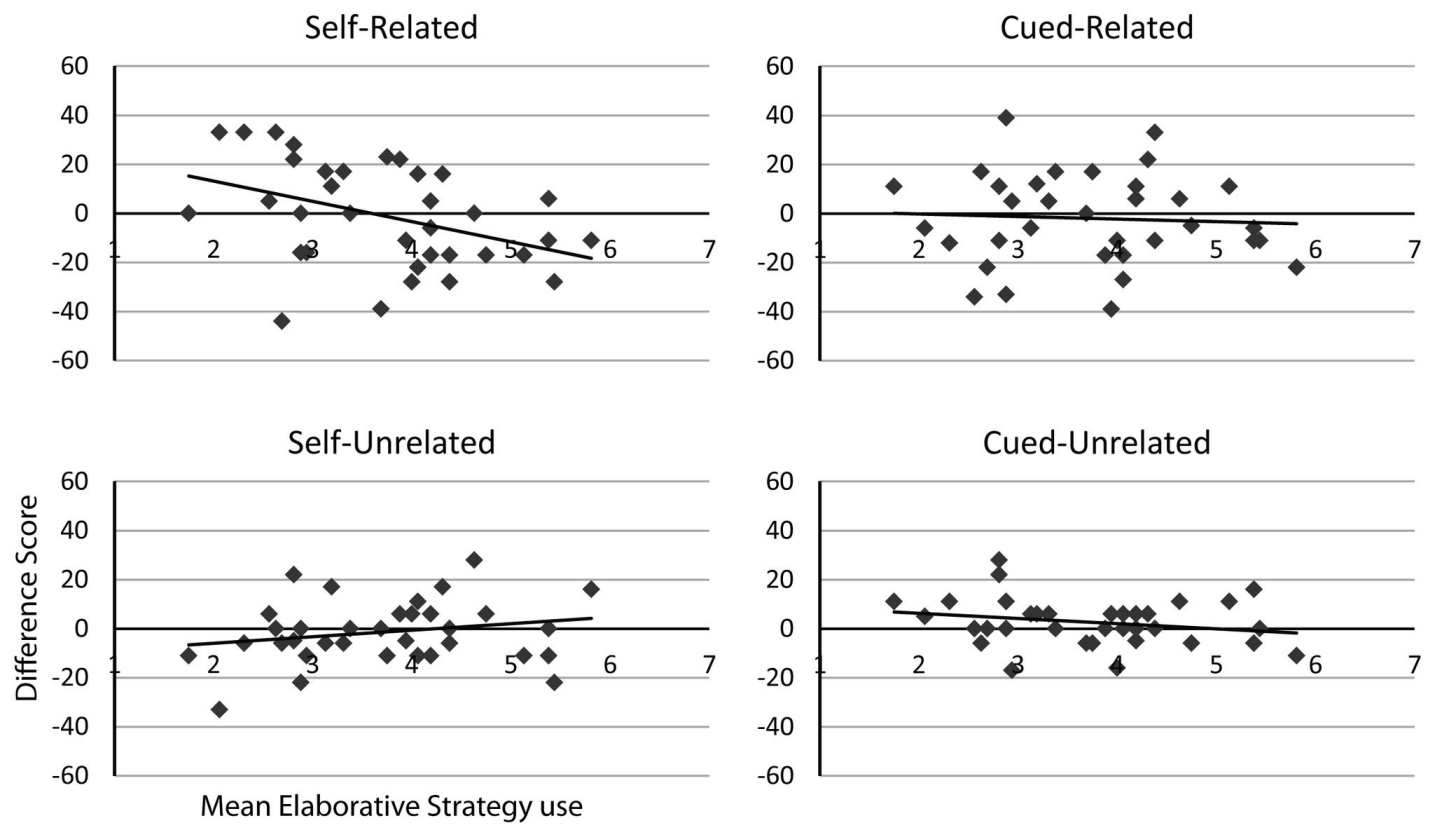
Scatter plots for difference scores of TMS (DLPFC - vertex) in cued recall performance and mean elaborative strategy use (questions 1 to 4 on the questionnaire, with participants indicating a range of strategy use for each question from 1, “never”, to 7, “always”), across the four experimental task conditions. A positive difference score indicated that participants had increased performance following DLPFC stimulation (compared to vertex), while a negative score indicates DLPFC stimulation during encoding reduced later cued recall performance. A significant correlation was only found for the self-related condition, using Spearman’s Rho.

We did not observe any significant differences across specific conditions despite our interaction, and also observed a significant interaction with strategy use. The relationship between the effects of rTMS and strategy use was therefore explored in greater details. A Pearson’s correlation (with a two-tailed significance test) was performed between the mean elaborative strategy use (represented as the means of questions 1 to 4, separately for each condition) and the difference score for each experimental condition, which resulted in a significant effect only in the self-related condition, Pearson’s correlation = -0.363, p = 0.034, all other conditions p > 0.1. Scatter plots of these correlations are presented in Figure 2, showing the relationship between strategy use and the effects of rTMS on memory encoding (as indexed by the difference score). Interestingly, the correlation in the self-related condition shows that in a sub-set of low strategy users, the application of rTMS to the left DLPFC actually improved memory performance relative to vertex stimulation.

To further explore the relationship between rTMS and strategy, a correlation analysis was performed on the difference score and each of the five questions specifically for that condition (e.g. we correlated the difference score for the self-initiated related condition to the response to Question 1 for self-initiated related, Question 2 for self-initiated related, etc). This analysis allows for a finer level of detail on specific memory strategies, as well as ensuring that the lack of significant correlations reported above is not masking an effect related to a specific encoding strategy. The results are presented in Table 2. Significant correlations between strategy use and the difference score were only observed in the self-initiated related condition, with significant correlations between the difference score and Question 1, rho = -0.345, p = 0.042, Question 2, rho = -0.432, p = 0.009, and Question 4, Rho = -0.412, p = 0.014, with a trending effect for Question 3, rho -0.286, p = 0.096. All other correlations were non-significant (all p > 0.1). This analysis further demonstrates that the effects of rTMS on memory were modulated by strategy use only in the self-initiated related condition.

**Table 2:** Correlations between difference scores and questionnaire results for each condition, with 95% confidence intervals.

**Cued Related**	**Q1**	**Q2**	**Q3**	**Q4**	**Q5**
Rho	-0.107	0.068	-0.052	0.041	0.03
	(-0.425, 0.235)	(-0.271, 0.392)	(-0.379, 0.286)	(-0.296, 0.369)	(-0.306, 0.360)
Sig. (2-tailed)	0.542	0.697	0.767	0.815	0.866
**Self related**	**Q1**	**Q2**	**Q3**	**Q4**	**Q5**
Rho	-.345*	-.432*	-0.286	-.412*	-0.204
	(-0.608, -0.013)	(-0.669, -0.115)	(-0.565, 0.052)	(-0.655, -0.091)	(-0.503, 0.139)
Sig. (2-tailed)	0.042	0.009	0.096	0.014	0.239
**Cued unrelated**	**Q1**	**Q2**	**Q3**	**Q4**	**Q5**
Rho	0.118	0.067	-0.256	-0.107	-0.039
	(-0.225, 0.434)	(-0.272, 0.392)	(-0.543, 0.084)	(-0.425, 0.235)	(-0.307, 0.298)
Sig. (2-tailed)	0.5	0.704	0.137	0.542	0.825
**Self unrelated**	**Q1**	**Q2**	**Q3**	**Q4**	**Q5**
Rho	0.166	0.232	0.213	0.155	-0.069
	(-0.177, 0.473)	(-0.110, 0.525)	(-0.129, 0.510)	(-0.188, 0.464)	(-0.393, 0.270)
Sig. (2-tailed)	0.34	0.179	0.22	0.375	0.692

* Correlation is significant at the 0.05 level (2-tailed).

Abbreviations: Q1 = question 1, Q2 = question 2, Q3 = question 3, Q4 = question 4, and Q5 = question 5. The correlations between difference scores are made with respect to questionnaire results from each question for that specific condition.

## Discussion

We performed a study using rTMS using a within-subjects cross-over design to test the hypothesis that the DLPFC plays a role in the self-initiation of elaborative encoding strategies, based on the findings of a previous fMRI study [32]. We used two encoding conditions: one cued participants towards elaborative encoding ('related?' instruction) while the other did not orient participants towards any specific encoding strategy or elaborative encoding ('memorize' instruction, or self-initiated). The critical condition in the study was the self-initiated related condition, in which elaborative strategy use (semantic analysis) improved memory, but was self-initiated. While the results of our MANCOVA analysis on the effects of rTMS on memory encoding showed a significant encoding condition by relatedness interaction, post hoc tests did not reveal any specific differences between experimental conditions. However, when we probed deeper using a correlational analysis, we observed an rTMS effect which was specific to the self-related condition, in line with our hypothesis. Interestingly, this effect was related to how much participants self-reported elaborative strategy use. Participants who made greater use of memory strategies tended to show a greater reduction in memory performance when rTMS was applied to the left DLPFC, suggesting that interfering with DLPFC function caused a reduction in effective encoding among high strategy users. In contrast, participants who made little use of memory strategies generally showed little effects of rTMS, or a facilitation of memory encoding following left DLPFC stimulation. A finer analysis, examining effects for specific memory strategies in each condition (Table 2), further supported this finding, in that again significant correlations between strategy use and the effects of rTMS were only observed in the self-related condition. This demonstrates that the observed relationship between strategy use and the rTMS difference score was not an artifact of utilizing overall strategy use (which may have obscured some relationships which were limited to specific memory strategies). While we did not observe the predicted overall effect of rTMS on the self-related condition, these findings do support the hypothesis that the left DLPFC plays a role in self-initiating elaborative strategy encoding as rTMS disrupted encoding only in the condition in which self-initiated encoding was most relevant, particularly in participants who made greater use of elaborative encoding strategies.

This difference in the effects of rTMS based on participant’s individual use of memory strategies is an important finding of this study. In another study using rTMS, right DLPFC stimulation during a recognition task has been found to reduce recognition for unfamiliar face-name pairs for people who reported using recognition memory strategies. In contrast, participants reporting not using strategies showed performance reduction following left DLPFC stimulation [31]. This suggests that strategy users and no strategy users may utilize different neural networks for task completion, at least for recognition memory. However, the results of the study by Manenti et al., should be interpreted with caution due to the small sample size (4 no strategy users and 10 strategy users). It nonetheless suggests that individual differences in strategy use can moderate the effects of rTMS on cognitive tasks. fMRI studies have also shown that individual differences in the pattern of brain activity may be related to individual strategy use during encoding [33,43]. The present results complement these findings, in that we found that participants who make minimal use of memory strategies showed a different effect of DLPFC compared to vertex stimulation than high strategy users in the self-related condition. This suggests that different individuals may be utilizing different neural networks during task performance, depending on the how they perform the task.

The observed findings that rTMS to the left DLPFC appears to enhance memory in low strategy users was an unexpected finding. We hypothesize that low strategy users are not utilizing the left DLPFC during encoding (during the self-related condition) in the same way, or to the same degree, as high strategy users (who show reduction in encoding performance following left DLPFC stimulation). One possible explanation, potentially supported by the study of Manenti et al. [31] described previously, is a laterality hypothesis. Low strategy users may be making greater use of the right DLPFC during encoding. Activity in the left DLPFC may be in competition with a network involving the right DLPFC. Therefore, when left DLPFC activity is disrupted, the alternate network (involving the right DLPFC) is freed of the competitive influences and facilitates memory performance in low strategy users. Additional studies using rTMS and/or fMRI may shine greater light on this issue.

An important implication of our results is that individuals may have substantially different responses to rTMS stimulation (as well as patterns of brain activity in neuroimaging studies), particularly for less structured tasks such as our self-initiated condition. We must therefore be careful in interpreting findings based on group means, which may reflect results from only a portion of participants or fail to show significance due to different patterns of activity across subgroups of participants. When participants who utilize different neural networks to accomplish the same task are mixed in a group analysis, it can be difficult to demonstrate consistent effects and draw strong conclusions. The best approach may be to move toward an experimental approach utilizing larger sampling sizes, and either separating participants into groups using techniques such as clustering analysis (which looks for patterns of results among sub-groups of participants), or collecting a large number of other measures (e.g. measures of cognitive ability or style, or task completion strategies).

Although several previous studies looking at DLPFC stimulation during encoding have reported a significant reduction in subsequent memory [24,25,27,30,41], we did not observe such findings. However, studies have not always observed reductions in memory following rTMS of encoding. For example, Rossi et al. [26] only found effects of rTMS when it was presented starting 500ms after stimuli onset, even though previous studies using earlier onset TMS showed decreases in encoding [24]. Sandrini et al. [27] only observed a reduction in memory performance when TMS was presented for unrelated word pairs, but not for related pairs. In working memory, rTMS has been observed to have a detrimental effect on performance only in the presence of task-irrelevant information, showing how changes in task design can eliminate significant rTMS effects. Stimulation of the left inferior frontal cortex during encoding has even been found to enhance later memory [44]. Strategy use may be one factor, among others (e.g. variations in memory test, such as cued or free recall, forced choice recognition, etc.), which accounts for variation in results across studies, in which small changes in the task or instructions may result in substantial changes in the effects of rTMS on encoding. Further research is necessary to fully understand what factors in study design and individual cognitive styles may have an impact on the results of rTMS studies, and networks of brain regions activated across tasks.

Our finding that rTMS had an effect in the self-related condition which was modulated by strategy use is in line with our hypothesis that the left DLPFC plays a role in self-initiation of encoding strategies. However, the effects were modulated by overall strategy use, suggesting that the relationship between left DLPFC and strategy use is not clear-cut. Instead, it suggests the possibility of differing neural networks across individuals, related to how much they utilize memory strategies. This may have particular relevance when considering clinical groups who may have a deficit in self-initiated strategy use, such as schizophrenia or Alzheimer’s Disease. Differences in neural activity in these individuals during cognitive tasks may reflect altered neural networks activated during tasks when individuals fail to utilize efficient strategies to perform a task.

## Supporting Information

Table S1 **Individuals participant’s response to each question in the strategy questionnaire, on a 7 point scale of 1 (not at all) to 7 (always).**
(XLSX)

Table S2 **Individual participant’s ques recall porportion of correct responses.** Participant excluded rom the analysis due to poor performance are highlighted in blue. TMS order indicates if the fisrt block of the experiment was DLPFC or vertex stimulation.
(XLSX)
